# Research on the Fusion of Dependent Evidence Based on Rank Correlation Coefficient

**DOI:** 10.3390/s17102362

**Published:** 2017-10-16

**Authors:** Fengjian Shi, Xiaoyan Su, Hong Qian, Ning Yang, Wenhua Han

**Affiliations:** School of Automation Engineering, Shanghai University of Electric Power, Shanghai 200090, China; shifengjian@mail.shiep.edu.cn (F.S.); qianhong@shiep.edu.cn (H.Q.); yangning@shiep.edu.cn (N.Y.); hanwenhua@shiep.edu.cn (W.H.)

**Keywords:** D–S evidence theory, dependent evidence, rank correlation coefficient

## Abstract

In order to meet the higher accuracy and system reliability requirements, the information fusion for multi-sensor systems is an increasing concern. Dempster–Shafer evidence theory (D–S theory) has been investigated for many applications in multi-sensor information fusion due to its flexibility in uncertainty modeling. However, classical evidence theory assumes that the evidence is independent of each other, which is often unrealistic. Ignoring the relationship between the evidence may lead to unreasonable fusion results, and even lead to wrong decisions. This assumption severely prevents D–S evidence theory from practical application and further development. In this paper, an innovative evidence fusion model to deal with dependent evidence based on rank correlation coefficient is proposed. The model first uses rank correlation coefficient to measure the dependence degree between different evidence. Then, total discount coefficient is obtained based on the dependence degree, which also considers the impact of the reliability of evidence. Finally, the discount evidence fusion model is presented. An example is illustrated to show the use and effectiveness of the proposed method.

## 1. Introduction

With the development of science and technology, in order to meet the higher accuracy and system reliability requirements, the information fusion filters for multi-sensor systems have been widely applied [1]. Various methods have been proposed for multi-sensor modeling and sensor data fusion [2,3,4,5,6,7], including night-vision image fusion [8], weighted measurement fusion and Unscented Kalman Filter [9], neural network models [10], fuzzy set theory [11], belief function theory [12], and so on.

The fusion of different uncertain data is an important research topic of modern intelligent multi-sensor systems. Among these methods, Dempster–Shafer evidence theory (D–S theory) has been investigated for many applications in multi-sensor information fusion due to its flexibility in uncertainty modeling [13,14,15]. D–S theory was first proposed by Dempster in 1967 [16], and further developed by Shafer in 1976 [17]. It can not only deal with imprecise information and uncertain information, but also deal with complimentary information and missing information [18,19,20,21]. Therefore, besides multi-sensor information fusion, D–S theory has also been investigated for applications in many fields such as fault diagnosis [22,23], pattern recognition [24,25,26,27,28], multi-source information fusion [29], multiple attribute decision making [30,31,32,33,34,35,36] and risk analysis [37,38,39,40].

In D–S theory, Dempster’s rule plays a vital part in the process of information fusion. However, there is an issue that limits the application of evidence theory. The classical evidence theory is based on the assumption that the evidence is independent of each other [41]. In practice, the dependence is more common. In other words, some elementary item of evidence will be counted twice without considering dependent evidence in the process of information fusion [42]. For example, one expert’s opinion may be affected by another expert’s opinion in an open decision making environment. In addition, there will be dependence between the evidence “grain production” and the evidence “natural disaster” in the agriculture risk analysis system [43]. A mistake will be generated if ignoring dependence between different evidence. To deal with dependent sources of information, many scholars proposed different methods [44]. The existing methods could be divided into two categories [45]: (1) improve the combination rule or (2) modify the original belief structure.

For the first category, the basic idea is to find a new evidence fusion method without considering dependence [45]. Some scholars have proposed their own combination rules. Cattaneo proposed the rules based on an assumption of minimal conflict rather than the independence assumption [46]. However, this method only analyzes the dependence between BBAs (Basic Belief Assignments) and fails to reveal the dependence between information sources. Destercke holds the view that the minimum rule of possibility theory could be generalized to the dependent evidence fusion [47,48]. However, in [18], the author thought this rule didn’t satisfy the fundamental evidence equation. In [42], the cautious rule of combination aiming at reliable sources of evidence and the bold disjunctive rule aiming at unreliable sources of evidence is proposed. Both of them satisfy commutative law, associative law and idempotent law. However, this combination rule was established in the canonical decomposition of BBA. Choenni proposed a dependent evidence fusion method using the idea of joint probability distribution of probability theory [49]. However, this method essentially deals with BBA as a discrete probability distribution and the fusion result is a couple of focal elements rather than BBA. Chebbah et al. proposed a new combination rule that takes consideration of sources’ degree of independence and they also suggest a method to quantify sources’ degree of independence [50].

For the second category, the basic idea is to reduce the repetitive computation of the dependent part of the information sources as far as possible [45]. In this category, there are two research ideas. The first research idea is based on the relevant source evidence model, which is first proposed in [51] by Smets. This paper holds that the reason why the two pieces of evidence are related is that they are obtained from the same source of evidence. The same source of evidence represents the correlation part between the pieces of evidence. Smets proposed a combination method in [51] based on the TBM (transferable belief model). Then, Xiao et al. proposed a combination rule based on the model in [51], and this rule is in the framework of D–S evidence theory. According to this theory, if we know the correlation part of two information sources, and the evidence of the correlation information source is available, this method is effective to deal with dependent evidence. However, this method is not reasonable as it does not care about the significance of the common evidence in some application systems. In addition, how to acquire the common evidence between two dependent pieces of evidence remains a question.

The second research idea is based on the discount evidence model. The main idea of this model is that dependent evidence shouldn’t be given the same weight as independent evidence in the process of information fusion since it provides less effective information [52]. The dependent evidence should be discounted in advance, and the discounting coefficients (or weight) are related to the degree of dependence. Guralnik et al. [53] presented a formal definition of algorithm dependency based on three criteria, i.e., method, sensors and features, and divided evidence into highly dependent, weakly dependent and independent evidence. Yager [54] proposed an interesting approach that makes use of a weighted aggregation of the belief structures where the weights are related to the degree of dependence. It is more practical to be used in real applications; however, how to define the degree of dependence is not addressed. To address this problem, Su et al. [43] presented a strategy of handling dependent evidence at a systematic level, which is able to capture both inner dependence (or interior relationship) and outer dependence (or exterior relationship). For inner dependence, they suggested using the analytic network process (ANP) to derive the degree of dependence. For outer dependence, they proposed a model based on the intersection of influencing factors identified during the information propagating and evaluating process. However, the method is subjective to some extent. For variables with a certain amount of historical data and samples, statistical methods can be used to measure the dependence among information sources. Su et al. [53] suggests using the Pearson correlation coefficient to represent the correlation between evidence. However, the Pearson correlation coefficient presents only a linear correlation between two variables, which is not always the case in real applications. In this paper, we proposed a method to measure the dependence between evidence based on rank correlation coefficient that could remove the limitation of Pearson correlation coefficient.

This paper is organized as follows. In Section 2, the preliminaries on D–S evidence theory, the definition of the discounted BBA and Spearman’s rank correlation coefficient are briefly introduced. In Section 3, the model based on Spearman’s rank correlation coefficient is proposed. In Section 4, an experiment is illustrated to show the rationality of this new method. Finally, the conclusions are given in Section 5.

## 2. Preliminaries

Some preliminaries are introduced in this section, including Dempster–Shafer evidence theory, the discounted evidence, Pignistic Probability Transformation and Spearman’s rank correlation coefficient.

### 2.1. Dempster–Shafer Evidence Theory

**Definition** **1.** *Let $\Theta =\{A_{1},A_{2},\cdots ,A_{N}\}$ be a finite nonempty set of N elements that are mutual and exhaustive, and we define *Θ* as Frame of Discernment [43]. Let P(Θ) be the power set composed of $2^{N}$ elements of *Θ*. The Basic Belief Assignment function (BBA) is defined as a mapping from the power set P(Θ) to a number between 0 and 1, m:P(Θ)→[0,1], which satisfies the following conditions [43]:*

(1) $$m\left(\emptyset \right)=0,\sum_{A\subseteq \Theta }m(A)=1,$$

*where m(A) denotes the Basic Belief Assignment of proposition A.*

**Definition** **2.** *(Dempster’s Rule) Let $m_{1},m_{2}\cdots m_{N}$ be N independent BBAs in the frame of Discernment of *Θ*. The result of their combination is denoted as $m=m_{1}⊕m_{2}⊕\cdots ⊕m_{N}$, and calculated as follows [16]:*

(2) $$\left\{\begin{array}{c} m(\emptyset )=0,\\ m\left(A\right)=K^{-1}\sum_{\cap A_{j}=A_{i}=1}\prod_{i=1}^{N}m_{i}\left(A_{j}\right), \end{array}\right.$$

*where K is normalizing factor, calculated as:*

(3) $$K=1-\sum_{\cap A_{j}=\emptyset }\prod_{i=1}^{N}m_{i}\left(A_{j}\right).$$

**Definition** **3.** *Let m be the BBA on *Θ* and α be the discount coefficient, α∈[0,1] the discounted BBA ${}^{\alpha }m$ defined as:*

(4) $${}^{\alpha }m=\alpha ⊗m:\left\{\begin{array}{c} {}^{\alpha }m(A)=\alpha m\left(A\right),\forall A\subset \Theta ,A\neq \Theta ,\\ {}^{\alpha }m\left(\Theta \right)=1-\alpha +\alpha m\left(\Theta \right). \end{array}\right.$$

**Definition** **4.** *(Pignistic Probability Transformation, PPT) To make a decision after BBA fusion results in acquisition, there are two methods: the first method is decision according to the BBA fusion results, the second method is translating the BBA fusion results to the probability and making decision. In the first method, the information loss may be large, and the second method helps to draw a more accurate result. Based on such consideration, Smets proposed the Pignistic Probability Transformation method [55]. Supposing m is BBA in *Θ*, let BetP be the Pignistic Probability distribution. The Pignistic Probability Transformation is defined as*

(5) $$\mathrm{Bet}P_{m}\left(\omega \right)=\sum_{A\subseteq \Theta ,\omega \in A}\frac{1}{\left|A\right|}\frac{m(A)}{1-m(\emptyset )},m\left(\emptyset \right)\neq 1,$$

*where |A| is the cardinality of A, and *∅* is denoted as the empty set.*

### 2.2. Spearman’s Rank Correlation Coefficient

There are all kinds of parameters to evaluate the dependent degree. The article [56] suggests using the Pearson correlation coefficient to represent the correlation between evidence. However, before using the Pearson correlation coefficient, it is necessary to assume that experiment data derived from normal distribution and was equidistant at least within the logical range. The rank correlation coefficient is a parameter-free measure for correlations that may be used to measure the level of agreement between two stochastic variables without making assumptions regarding the parametric structure of the probability distribution of the variables. The rank correlation coefficient, a parameter independent of the distribution, was proposed by Sperman in 1904 and used to measure the correlation between the two variables [57,58].

The basic idea of the Spearman rank correlation coefficient is to use the rank of the variable instead of the specific data for statistical inference [59]. Suppose that the variables *x* and *y* have *n* samples (measured values) denoted as $x_{i}$, $y_{i}$, where *i* = 1, 2, ⋯ *n*. Sorting the sample data from large to small (or from small to large), let $x_{i}^{\prime }$, $y_{i}^{\prime }$ be the position of original data $x_{i}$, $y_{i}$ after arrangment. The Spearman rank correlation coefficient is defined as

(6) $$r_{s}=\frac{\sum_{i=1}^{n}\left({x^{\prime }}_{i}-{\bar{x}}^{\prime }\right)\left({y^{\prime }}_{i}-{\bar{y}}^{\prime }\right)}{\sqrt{\sum_{i=1}^{n}{\left({x^{\prime }}_{i}-{\bar{x}}^{\prime }\right)}^{2}\sum_{i=1}^{n}{\left({y^{\prime }}_{i}-{\bar{y}}^{\prime }\right)}^{2}}}=1-\frac{6\sum_{i=1}^{n}d_{i}^{2}}{n(n^{2}-1)},$$

where $-1\leq r_{s}\leq 1$, the $|r_{s}|$ is growing with x,y closer and closer to the strict monotonic function. $r_{s}=1$ represents x,y becoming a strictly monotone increasing function and $r_{s}=-1$ represents x,y becoming a strictly monotone decreasing function. If $r_{s}=0$, x,y have no relevance to the distinct monotonic function.

## 3. Proposed Method

### 3.1. The Framework of the Proposed Method

**Figure 1:**
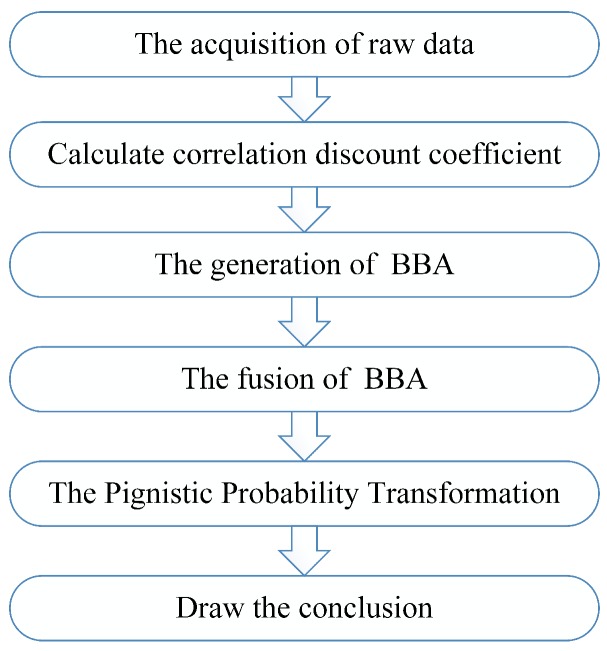
Flowchart of the proposed method.

In this section, the method of handling dependent evidence is given in detail in order to fuse dependent sensor data properly. A flowchart of the proposed method is given in Figure 1. From Section 1, we can know that there are two basic directions to handle dependent evidence. One is to modify the Dempster’s combination rule, finding a new evidence fusion method without considering dependence. The other is to reduce the repetitive computation of the dependent part of the information sources as far as possible. Here, we adopt the latter method to establish our evidence fusion model. First, collect sensor data as the raw data to generate BBA. Then, analyze the association between every two sensor information sources and calculate the correlation discount coefficient based on the analysis of sensor data. In addition, fuse the discounted BBA according to Dempster’s combination rule. Finally, a decision conclusion is making by using the method of Pignistic Probability Transformation proposed in [55].

### 3.2. The Generation of the Correlation Discount Coefficient

**Figure 2:**
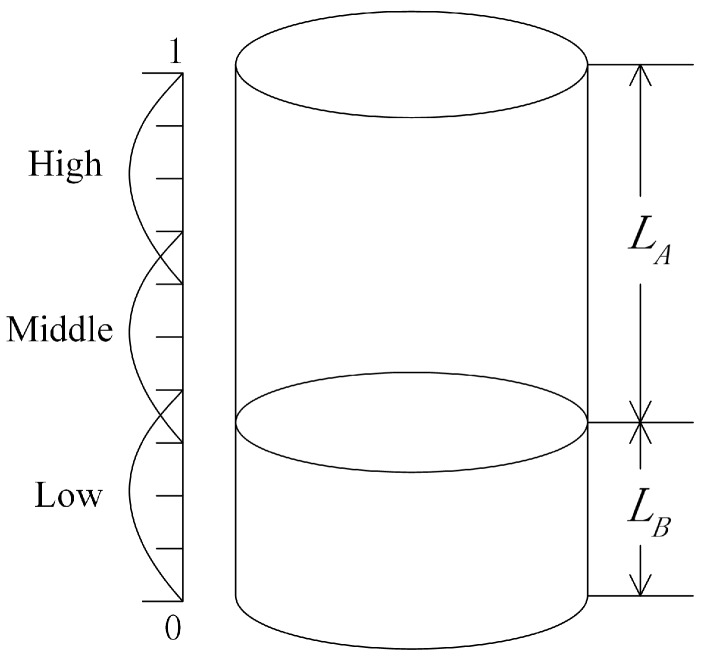
Measurement of the water level.

Thinking of the need to experience the construction and fusion of BBA from the sensor information source and the final decision making, the analysis of the relevance of the evidence should begin with the initial information source, as the real application involves multi-sensor sources of information, and the relationship between the sensor information source is complex. In order to find a simple and effective representation, this paper adopts the following method:

**Step 1:**  Calculate the Spearman rank correlation coefficient between every two sensor information sources according to Equation (6).
**Step 2:**  The correlation between the two sensor information sources can be divided into positive correlation and negative correlation. In other words, rank correlation coefficients are positive or negative, and negative correlation evidence can be regarded as conflict evidence. Calculate the dependence degree $d_{sx,sy}$ between two information sources:
  (7) $$d_{sx,sy}=\left|r_{sx,sy}\right|.$$
  Here, $d_{sx,sy}$ is defined as the absolute value of $r_{sx,sy}$ because the positive correlation or negative correlation does not substantially affect the fusion result for the information fusion system based on the D–S evidence theory. To illustrate this problem, there is an example:
  Suppose that there are two sensors A and B to qualitatively measure the water level. The recognition framework is defined as Θ={high,middle,low}. As shown in Figure 2, sensor A measures the distance between the top of the well and water surface, denoted as $L_{A}$, while sensor B measures the distance between the bottom of the well and water surface, denoted as $L_{B}$.
  Assume that the depth of this well is 1. The interval [0, 0.4], [0.3, 0.7] and [0.6, 1], respectively, represent the statuses of low water level, middle water level and high water level. By analysing the geometric relationship, $L_{A}$ and $L_{B}$ satisfy the follow equation:
  (8) $$L_{A}=-L_{B}+1.$$
  That is, $L_{A}$ and $L_{B}$ have a strictly negative correlation relationship, $r_{A,B}=-1$. However, $L_{A}$ and $L_{B}$ may have different values for the same water level, and they established the same BBA for the water level. For example, when $L_{A}=0.9$, $L_{B}=0.1$, both of the sensors establish the same BBA:
  (9) m({low})=1.
  In addition, when $L_{A}=0.35$, $L_{B}=0.65$, both of the sensors establish the same BBA:
  (10) m({middle},{high})=1.
  In other words, the positive correlation or negative correlation does not substantially affect the fusion result for the information fusion system based on the D–S evidence theory.
  Supposing that there are M sensor sources, we can then establish dependency matrix *D*:
  (11) $$D=\left[\begin{array}{cccc} d_{S1,S1} & d_{S1,S2} & \cdots & d_{S1,SM}\\ d_{S2,S1} & d_{S2,S2} & \cdots & d_{S2,SM}\\ \vdots & \vdots & \ddots & \vdots \\ d_{SM,S1} & d_{SM,S2} & \cdots & d_{SM,SM} \end{array}\right].$$
**Step 3:**  Calculate the total dependence degree of each sensor information source. The total dependence degree of $S_{i}$ is defined as
  (12) $$T_{Si}=\sum_{k=1}^{M}d_{Si,Sk}.$$
**Step 4:**  Considering that the evidence with strong relevance should be given a smaller discount factor, the correlation discount coefficient is defined as
  (13) $$\alpha_{Si}=\frac{1}{T_{Si}}.$$

### 3.3. Reliability Assessment

In a multi-sensor information fusion system, the global system performance is closely related to each sensor’s reliability. The reliability of the information source refers to the correct rate obtained by direct decision by the information source. The higher reliability evidence helps to draw a more accurate result in the decision-making process. In addition, the higher reliability evidence should be given larger weight in evidence fusion. For the target recognition, it is assumed that there are *N* groups of training data to establish BBA by $S_{i}$ and *M* of them have correct classification results. The reliability of the information source $S_{i}$ is defined as

(14) $$\beta_{Si}=\frac{M}{N}.$$

### 3.4. The Fusion of Dependent Evidence

On the basis of the above analyses, the total discounting coefficient of evidence from information source $S_{i}$, represented by $\omega_{Si}$, can be defined as

(15) $$\omega_{Si}=\alpha_{Si}\beta_{Si}.$$

Suppose that BBA $m_{S1},m_{S2},\cdots m_{SM}$ is established by $S_{1},S_{2},\cdots S_{M}$, whose total discounting coefficient is $\omega_{S1},\omega_{S2},\cdots \omega_{SM}\in \left[0,1\right]$. The formula of dependent evidence fusion is as follows:

(16) $$m={}^{\omega_{S1}}ms_{1}⊕{}^{\omega_{S2}}ms_{2}⊕\cdots ⊕{}^{\omega_{SM}}ms_{M},$$

where ${}^{\omega_{Si}}ms_{i}$ represents discount calculation with discounting coefficient $\omega_{Si}$. (see Equation (4)).

## 4. Experiment and Discussion

In this paper, Iris Dataset from the machine learning database [60] is used as the data source of the experiment. The dataset contains three types of irises, such as Setosa, Versicolour and Virginica, and each group of irises contains 50 sets of data samples. Each group has four different attributes: SL (Sepal Length), SW (Sepal Width), PL (Petal Length) and PW (Petal Width). These four different attributes can be used as four kinds of information sources to construct BBA.

### 4.1. Possible Application of the Proposed Evidence Fusion Model

The dependent evidence fusion method in this paper could effectively deal with uncertain information fusion issues existing in the real world. One of the applications of this method could be target recognition. For example, to recognize the enemy aircraft, which could be in the form of a bomber, the Air Early Warning plane or fighter plane, we could first acquire information such as airborne radar signal, infrared signal or the electronic support measure (ESM) information. Different types of enemy aircraft usually have different features in the information. Thus, the type of aircraft could be recognized based on the acquired information. The recognition rate can be improved by combining information from different sensors. The dependence among the sensors is also considered in the proposed method. Similar to the iris example, the airborne radar signal, infrared signal or the ESM information are associated with the iris attributes SL, SW, PL or PW, and the types of enemy aircraft are associated with the iris types Setosa, Versicolour or Virginica.

Another possible application could be fault diagnosis. For example, in a power transformer fault diagnosis system, we want to recognize different fault types including transformer winding, transformer core, arc discharge or transformer insulation aging, and so on. In addition, the corresponding fault symptoms including transformer core earth current, insulation resistance or other symptoms could be acquired to establish different BBAs. Different fault symptoms may be dependent and the fusion method proposed in this article can handle this problem. In other words, the dependent information fusion model based on rank correlation coefficient could be investigated for applications in many fields.

### 4.2. Experimental Method

**Figure 3:**
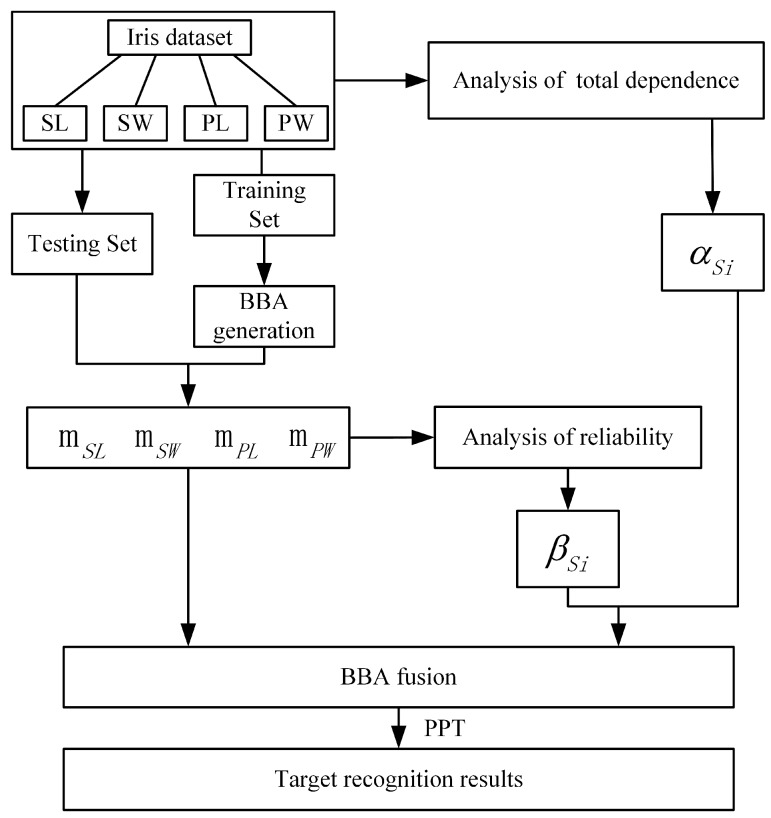
The main procedure of the proposed method to recognize iris class.

The main procedure of the proposed method to recognize iris class is shown in Figure 3.

In the experiment, some samples were randomly selected as the training set, and the remaining samples were used as test set. The steps are as follows:

**Step 1:**  Calculate the correlation discount coefficient $\alpha_{Si}$ of four attributes. Refer to Section 3.2.
**Step 2:**  Build BBA. Four BBAs were established for SL, SW, PL and PW. The BBA was established according to the article [61].
**Step 3:**  Calculate reliability coefficient $\beta_{Si}$, and refer to Section 3.3.
**Step 4:**  Calculate the total discounting coefficient of four attributes according to Equation (15).
**Step 5:**  Model testing. Using the test data, we assume for four cases that the evidence is independent from each other, only considering the dependence between the evidence, only considering the reliability of the evidence, considering both dependence and reliability, and then calculating the recognition accuracy. Then, we increase the proportion of training set data, and repeat the above experiment. In the four cases, the fusion rule as follows:

Group 1: Assume that the evidence is independent from each other
  (17) $$m=ms_{1}⊕ms_{2}⊕\cdots ⊕ms_{M}.$$
Group 2: Only considering the dependence between the evidence
  (18) $$m={}^{\alpha_{S1}}ms_{1}⊕{}^{\alpha_{S2}}ms_{2}⊕\cdots ⊕{}^{\alpha_{SM}}ms_{M}.$$
Group 3: Only considering the reliability of the evidence
  (19) $$m={}^{\beta_{S1}}ms_{1}⊕{}^{\beta_{S2}}ms_{2}⊕\cdots ⊕{}^{\beta_{SM}}ms_{M}.$$
Group 4: Considering both dependence and reliability
  (20) $$m={}^{\omega_{S1}}ms_{1}⊕{}^{\omega_{S2}}ms_{2}⊕\cdots ⊕{}^{\omega_{SM}}ms_{M}.$$

The method of decision-making after fusion is based on the Pignistic Probability Transformation (PPT) proposed in [55].

### 4.3. Experimental Procedure

In this section, the correlation of iris data sets is performed. The main procedures are as follows:

**Step 1:**  The first step is to analyse the dependence among SL, SW, PL, and PW attributes. Rank correlation coefficients among attributes of iris data sets are shown in Table 1.
  Then, we can establish dependency matrix D:
  $$D=\left[\begin{array}{cccc} 1.0000 & 0.1595 & 0.8814 & 0.8344\\ 0.1595 & 1.0000 & 0.3034 & 0.2775\\ 0.8814 & 0.3034 & 1.0000 & 0.9360\\ 0.8344 & 0.2775 & 0.9360 & 1.0000 \end{array}\right].$$
  Calculate the total dependence degree of each attribute as Equation (12). Results are as follows:
  $$T_{SL}=2.8753,T_{SW}=1.7404,T_{PL}=3.1208,T_{PW}=3.0479.$$
  Considering that the evidence with strong relevance should be given a smaller discount factor, the correlation discount coefficient is calculated as Equation (13). The results are as follows:
  $$\alpha_{SL}=0.3478,\alpha_{SW}=0.5746,\alpha_{PL}=0.3204,\alpha_{PW}=0.3281.$$
**Step 2:**  Build BBA. Four BBAs were established for SL, SW, PL and PW. The BBA was established according to the article [61].
**Step 3:**  Calculate reliability coefficients of the four attributes as Equation (14). The results are as follows:
  $$\beta_{SL}=0.7267,\beta_{SW}=0.5467,\beta_{PL}=0.9533,\beta_{PW}=0.9600.$$
**Step 4:**  The total discounting coefficient of the four attributes can be calculated as Equation (15). The results are as follows:
  $$\omega_{SL}=0.2527,\omega_{SW}=0.3141,\omega_{PL}=0.3054,\omega_{PW}=0.3150.$$
  After normalization, the total discounting coefficient of the four attributes is
  $$\omega_{SL}=0.8022,\omega_{SW}=0.9971,\omega_{PL}=0.9695,\omega_{PW}=1.$$
**Step 5:**  Model testing. Based on the above calculation, we begin to test our evidence fusion model. The detailed steps are shown in Section 4.2.
**Step 6:**  Then, we calculate the confidence interval of this experiment. For each piece of evidence, we can acquire a BBA, and suppose its recognition result is A. Then, the Belief function (Bel(A)), defined as a sum of the mass probabilities of all the proper subsets of A, is calculated as follows:
  (21) $$Bel\left(A\right)=\sum_{B\subseteq A}m\left(B\right).$$

In addition, the Plausibility function ( Pl(A) ), defined as maximum belief of A, is calculated as follows:

(22) $$Pl\left(A\right)=\sum_{B\cap A\neq \emptyset }m(B).$$

Then, the Belief Interval is defined as $\left[Bel\left(A\right),\;Pl\left(A\right)\right]$. Here, calculate the average Bel and Pl of each proportion of testing data.

### 4.4. Results and Analysis

**Figure 4:**
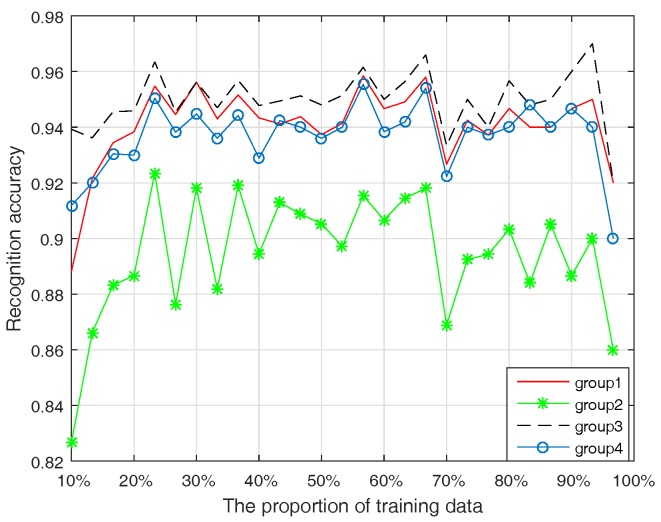
Average classification recognition accuracy in four cases.

In four cases, the average classification recognition accuracy test results are shown in Figure 4.

**Figure 5:**
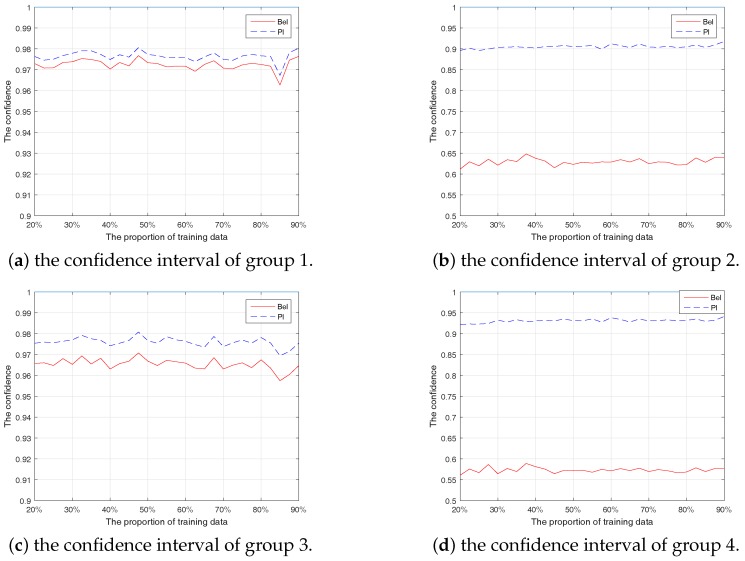
The confidence intervals of the experiments.

The confidence interval of this experiment as shown in Figure 5.

Comparing Group 1 with Group 3 (or Group 2 with Group 4), it is obvious that the recognition accuracy is significantly improved when considering reliability. However, comparing Group 1 with Group 2 (or Group 3 with Group 4), the recognition accuracy is decreased. In addition, further research shows the reliability coefficients of the four attributes and the correlation coefficients in Table 2.

From Table 2, the reliability coefficient of PW or PL is large, indicating higher reliability. However, the correlation coefficient of PW or PL is small, indicating higher dependence of these two attributes in the whole system. The reliability coefficient of SW is small (lower reliability), while its correlation coefficient is the largest (the most independent attribute in the whole system). In this experiment, a higher recognition accuracy was obtained when using PW or PL attributes. However, there is strong dependence between PW and PL, and the higher recognition accuracy of PW or PL attributes is actually unreasonable for repeating fusion of two similar pieces of evidence. The recognition accuracy will be decreased when considering the effect of dependent evidence. On the other hand, the recognition accuracy will be below actual accuracy if two dependent pieces of evidence that both have low recognition accuracy are fused. In this case, the total recognition accuracy will increase when considering dependent evidence. In the next section, a case study is used to show the effectiveness of the proposed method.

### 4.5. Further Study

A case study is used to show the effectiveness of the proposed method. Assume that the framework is Θ={A,B,C}, and four independent pieces of evidence are R1, R2, R3 and R4. In this case, the correct recognition result is A. As are shown in the following table, and the pieces of evidence R1, R2, and R3 could draw the correct result; however, evidence R4 draws the wrong result (Table 3).

**Case1:** Combining these four independent pieces of evidence according to the traditional Dempster’s rule:

$$m=m_{R1}⊕m_{R2}⊕m_{R3}⊕m_{R4}$$

after PPT, the probability is

p(A)=0.7834,p(B)=0.2062,p(C)=0.0104.

The recognition result is A, which is correct by combining four independent pieces of evidence.

**Case2:** Let us consider another condition: we have the fifth piece of evidence R5, which is the same as R4, that is, R5:m(AC)=0.15,m(B)=0.55,m(BC)=0.3. Apparently, R4 is totally dependent on R5, and there may be a mistake if we combine these five pieces of evidence regardless of their association; for example:

$$m=m_{R1}⊕m_{R2}⊕m_{R3}⊕m_{R4}⊕m_{R5}.$$

after PPT, and the probability is

p(A)=0.3942,p(B)=0.5880,p(C)=0.0178.

The recognition result is B; therefore, using traditional Dempster’s rule without considering dependent can draw a wrong result. The reason is that one of the pieces of evidence is totally dependent on another piece of evidence, which means that one of the pieces of evidence has been counted twice.

**Case3:** By considering the dependent evidence based on the method proposed in Section 3, the correlation discount coefficient could be given as follows:

$$\alpha_{R1}=1,\alpha_{R2}=1,\alpha_{R3}=1,\alpha_{R4}=0.5,\alpha_{R5}=0.5.$$

Then, the fusion rule is

$$m={}^{\alpha_{R1}}m_{R1}⊕{}^{\alpha_{R2}}m_{R2}⊕{}^{\alpha_{R3}}m_{R3}⊕{}^{\alpha_{R4}}m_{R4}⊕{}^{\alpha_{R5}}m_{R5}.$$

The final result is

p(A)=0.8881,p(B)=0.1068,p(C)=0.0051.

The recognition result is A. Thus, the proposed dependent evidence fusion model could improve the decision-making result especially in the fusion of low recognition evidence.

## 5. Conclusions

With the rapid development of artificial intelligence, the acquisition of information has increasing importance. In industrial applications, the multi-sensor information fusion system based on Dempster–Shafer evidence theory plays a more and more important role in information collection and decision-making. However, the classical evidence theory assumes that the evidence is independent from each other, which is often difficult to establish in practice. To address this issue, this paper analyzes the present researche about dependent evidence fusion. Comparing with all kinds of correlation measurement methods, we select the Spearman’s rank correlation coefficient as the metric to measure dependence existing in evidence. Rank correlation coefficient is a parameter-free measure for correlations, which may be used to measure the level of agreement between two stochastic variables without making assumptions regarding the parametric structure of the probability distribution of the variables. Then, a dependent evidence fusion model based on rank correlation coefficient is established. Finally, an experiment is developed to verify the effectiveness of this model based on iris data sets. Experiment results suggest that considering reliability will improve accuracy of decision-making and considering dependent evidence helps draw more reasonable and robust conclusions. In other words, it is necessary to consider the influence of dependent evidence in information fusion to gain a more credible result.

## Figures and Tables

**Table 1:** Correlation coefficients among attributes.

Attribute	SL	SW	PL	PW
**SL**	1.0000	−0.1595	0.8814	0.8344
**SW**	−0.1595	1.0000	−0.3034	−0.2775
**PL**	0.8814	−0.3034	1.0000	0.9360
**PW**	0.8344	−0.2775	0.9360	1.0000

**Table 2:** The reliability coefficient and correlation coefficient of four attributes.

Coefficient	SL	SW	PL	PW
**reliability coefficient**	0.7267	0.5467	0.9533	0.9600
**correlation coefficient**	0.3478	0.5746	0.3204	0.3281

**Table 3:** Four pieces of evidence and their recognition results.

Item	BBA	PPT	Recognition Result
**R1**	m(A)=0.5,m(B)=0.2,m(AC)=0.3	p(A)=0.65,p(B)=0.2,p(C)=0.15	A
**R2**	m(A)=0.55,m(B)=0.15,m(ABC)=0.3	p(A)=0.65,p(B)=0.25,p(C)=0.1	A
**R3**	m(A)=0.61,m(AB)=0.35,m(AC)=0.04	p(A)=0.805,p(B)=0.175,p(C)=0.02	A
**R4**	m(AC)=0.15,m(B)=0.55,m(BC)=0.3	p(A)=0.075,p(B)=0.7,p(C)=0.225	B
